# An epistemology for democratic citizen science

**DOI:** 10.1098/rsos.231100

**Published:** 2023-11-15

**Authors:** Johannes Jaeger, Camille Masselot, Bastian Greshake Tzovaras, Enric Senabre Hidalgo, Mordechai (Muki) Haklay, Marc Santolini

**Affiliations:** ^1^ Complexity Science Hub (CSH) Vienna, Josefstädter Straße 39, 1080 Vienna, Austria; ^2^ Université Paris Cité, Inserm, System Engineering and Evolution Dynamics, 75004 Paris, France; ^3^ Learning Planet Institute, 8 bis Rue Charles V, 75004 Paris, France; ^4^ Institut d’Études Avancées de Paris, 17 quai d'Anjou, 75004 Paris, France; ^5^ Université Paris-Saclay, 3 rue Joliot Curie, 91190 Gif-sur-Yvette, France; ^6^ Universitat Oberta de Catalunya, Rambla del Poblenou 156, 08018 Barcelona, Spain; ^7^ Department of Geography, University College London, Gower Street, London WC1E 6BT, UK

**Keywords:** citizen science, epistemology, perspectival realism, process thinking, deliberative practice, project assessment

## Abstract

More than ever, humanity relies on robust scientific knowledge of the world and our place within it. Unfortunately, our contemporary view of science is still suffused with outdated ideas about scientific knowledge production based on a naive kind of realism. These ideas persist among members of the public and scientists alike. They contribute to an ultra-competitive system of academic research, which sacrifices long-term productivity through an excessive obsession with short-term efficiency. Efforts to diversify this system come from a movement called democratic citizen science, which can serve as a model for scientific inquiry in general. Democratic citizen science requires an alternative theory of knowledge with a focus on the role that diversity plays in the process of discovery. Here, we present such an epistemology, based on three central philosophical pillars: perspectival realism, a naturalistic process-based epistemology, and deliberative social practices. They broaden our focus from immediate research outcomes towards cognitive and social processes which facilitate sustainable long-term productivity and scientific innovation. This marks a shift from an industrial to an ecological vision of how scientific research should be done, and how it should be assessed. At the core of this vision are research communities that are diverse, representative, and democratic.

## 1. Introduction

The way we do science and the role of science in society are rapidly changing. It has been a long time since science was carried out by a small and exclusive elite of independently wealthy gentlemen scientists. Much has changed for the better: a steady and massive increase in global science funding has enabled the establishment, professionalization, internationalization, and diversification of many flourishing research fields. At the same time, however, the increased flow of money through the system has also resulted in intensified pressures of various kinds, fragmentation, specialization, and the commodification of science. Since the Second World War and the emergence of ‘Big Science’, doing research has become more and more like a factory business—a production line based on fine-tuned division of labour—geared and optimized towards the efficient generation of tangible results. With the increasing dominance of market-oriented approaches—due to the rise of neoliberalism since the 1980s—an expectation has arisen that science will respond to market mechanisms with an emphasis on efficiency, measurable productivity, and the expectation that even basic research should yield innovation and foster economic activity down the line.

Efficiency is certainly not a bad thing *per se*, especially considering the range of urgent problems facing humanity in the twenty-first century. And yet, an obsessive focus on efficiency can hamper long-term productivity when the free exploration of ideas is pitted against the expectation of short-term local returns, preferably in terms of monetary gains and technological applications (e.g. [1,2]). On top of this, with the growth of inequalities in Western societies and the focus of scientists on the needs of government and corporations, many people are realizing that a significant part of scientific research has become detached from the harsh realities outside the ivory tower. This goes hand-in-hand with a polarizing trend of interpreting scientific insights along partisan lines on either end of the political spectrum, leading to increasing suspicion against such politicized research.

In light of this complicated situation, it is highly necessary for us to reconsider the way we do science in our present socio-political context. Humanity is currently living through a period of profound crisis, affecting our ecological sustainability, the stability of our socio-economic and political systems, and—at the bottom of it all—our ability to make sense of a fast-changing, complex, and highly interconnected world. More than ever before, we need trustworthy knowledge, as well as accessible and reliable means to transmit it to others. We need scientific knowledge for the public good, based on open inquiry—a science which serves the interests of all, which asks and researches questions that are important to the whole of society, and listens to marginalized voices. But how can we ensure the free pursuit of knowledge, without reverting to the elitist gentlemen-science of yore, without neglecting the fact that we urgently need actionable solutions for pressing real-world problems? How can we restore the public's trust in science without losing academic freedom, without rendering it vulnerable to populist political whims? In essence, how can we achieve *a true democratization of science*—a democratization of the process of inquiry itself (see [3] for an early exposition of this problem; recently reviewed in [4]), but also of the relationship between professional scientists and those who are affected by their work? More specifically, how can we achieve democratization without jeopardizing the independence, authority, and long-term productivity of science?

Here, we look at this fundamental question from a philosophical (but also practical) angle. *Citizen science*, broadly defined, is an umbrella term which includes any scientific project in which individuals or communities who are not professional researchers (and may not even have formal scientific training) participate in research design, data collection, and/or data analysis [5–7]. Citizen science denotes a way of organizing scientific inquiry, and in some cases a methodology to carry out research, rather than a specific type of research project [8]. In fact, citizen science projects are extremely diverse. On one end of the spectrum, there are those that are driven by traditional scientific authorities and follow a model of traditional research, but collect data through gamified crowdsourcing (as in the FoldIt project on protein folding: http://fold.it), or community-wide observations and annotation efforts (e.g. Galaxy Zoo, analysing images from different space surveys: https://www.zooniverse.org/projects/zookeeper/galaxy-zoo; these and other examples are reviewed in [8,9]). In this type of citizen science, research design, data analysis and quality control remain exclusively in the hands of professional scientists, and although evidence shows that participants learn through taking part in a study, their scientific education is not always a major focus.

On the other end of the spectrum, there are community-based projects that are not aimed at new conceptual breakthroughs or theoretical frameworks. Instead, they are using applied science to address local concerns: monitoring and improving water or air quality in a community (e.g. [10]). Other examples of such community-driven projects with an environmental focus can be found on https://publiclab.org, https://smartcitizen.me and https://safecast.org. Such projects are often driven by nonscientists. They are often gathered under the umbrella term *community science* to distinguish them from traditional citizen science projects such as those described in the previous paragraph [5].

In the context of our discussion here, we are most interested in a kind of citizen science that lies between these two extremes. In particular, we are interested in projects that actively involve a broad range of participants in project design, data analysis, *and* quality monitoring, with the triple aim of generating new scientific knowledge, of teaching participants about science, and finding solutions to a local or regional problem. We take this kind of *democratic, participatory, social-movement-based* or *socially engaged citizen science* as an ideal worth aspiring to (as do others; see [11–14], but also [4] for a more critical assessment)*.* More generally, we believe that it serves as a good model for the kind of reforms we need for the democratization of scientific research in general, beyond the specific domain of citizen science.

Much has been written about the historical, political and sociological aspects of democratic citizen science (e.g. [12,14,15]). It differs significantly from traditional academic research in its goals, values, attitudes, practices and methodologies. It is a bottom-up approach that aims to democratize research processes through deliberative practices (see §5). Apart from its focus on the process of inquiry, democratic citizen science has a number of obvious advantages when considered from a political or ethical point of view. It not only taps into a large base of potential contributors [9,16], generally incurring a relatively low amount of costs per participant, but also attempts to foster inclusion and diversity in scientific communities (see [5] for a critical discussion), opens a channel of communication between scientists and nonscientists, and provides hands-on science education to interested citizens. Democratic citizen science can help to address the problems of *undone science*—important areas of inquiry which are neglected due to competing political agendas [17]—and of *epistemic injustice*—inequalities in the accessibility and distribution of scientific knowledge [18]. It aims to bring scientific knowledge to all those who most urgently need it, rather than only those few who provide the bulk of the funding. Its open design is intended to increase the reproducibility, adequacy, and robustness of the scientific results it generates, and to promote collaboration over competition in the process of inquiry. Last but not least, with its bottom-up approach, it challenges the hierarchical nature of scientific knowledge, which has often been taken for granted ever since discussions about science and democracy first arose (e.g. [19,20]).

All these benefits, of course, rely on the implementation and monitoring of procedures and protocols that ensure good scientific practice, management and data quality control. Other challenging aspects of democratic citizen science are its relatively low per-person productivity (compared to that of full-time professional researchers who generally require less instruction and supervision), and an increased complexity in project management—especially if citizen scientists are not merely employed for data collection, but are also involved in project design, quality monitoring as well as the analysis and interpretation of results. At the same time, this complexity and deliberative nature holds benefits such as reduction of mistakes, ability to replicate and verify information and design, and use of transdisciplinary insights that come from participants.

Beyond these practical considerations, there is a more philosophical dimension to democratic citizen science that has received surprisingly little attention so far (see [13,14,16,21–23] for a number of notable exceptions). It concerns the theory of knowledge, the kind of *epistemology* able to describe, analyse and support the efforts of democratic citizen science. In other words, *to assess the practicality, usefulness, ethics, and overall success of democratic citizen science, we need to take seriously the kind of knowledge it produces, and the way by which it produces that knowledge*. It is this largely unexamined epistemological aspect of citizen science that we want to analyse in this paper.

To precisely pinpoint and highlight the differences between knowledge production by democratic citizen science compared to traditional academic research, we make use of an argumentative device: we present an epistemology ideally suited for citizen-science projects of the democratic kind by contrasting it with a very traditional view of scientific epistemology. Our intention is not to build a straw man argument, or to paint an oversimplified black-and-white picture of (citizen) science. We are very well aware that the epistemic stances of many scientists and citizens are much more sophisticated, nuanced, and diverse than those depicted here (e.g. [24]). However, even though the philosophy of science may have moved on, many practicing scientists and stakeholders of science still do retain remnants of a decidedly old-fashioned view of science, which we will call *naive realism* (ibid.)*.* In most cases, this view is not explicitly formulated in the minds of those who hold it and its assumptions and implications remain unexamined. Nor does this view amount to a consistent or systematic philosophical doctrine. Instead, naive realism consists of a set of more or less vaguely held convictions, which often clash in contradictions, and leave many problems concerning the scientific method and the knowledge it produces unresolved. Yet, somehow, these ideas tenaciously persist and hold a firm grip on what we—as communities of scientists, stakeholders and citizens—consider to be the epistemic goal and the societal role of scientific research.

It should be quite clear that the persistence of naive realism is not a purely theoretical or philosophical problem. One of its major theoretical implications is that it treats the whole world as a machine, an engineered clockwork, to be understood in purely formal and mechanistic terms. One of its major practical implications concerns the way we assess the success of research projects (see [25] for an historical overview). What we value crucially depends on how we define the epistemic (and non-epistemic) goals of science, and what we consider high-quality scientific knowledge. We will argue below that naive realism leads to a system of incentives which is excessively focused on misguided notions of accountability and short-term productivity—in particular, the efficient generation of measurable research output [26]. We could call this *the industrial model of doing science*, since it treats research as a system of mechanical production, which must be put under tight, top-down control.

In such an industrial system, projects of democratic citizen science are at a fundamental disadvantage. Standard assessment practices do not do justice to the diversified ways by which such projects generate knowledge and other benefits for the participants and stakeholders involved [27,28]. Even more importantly, democratic citizen science cannot compete with traditional academic science in terms of production efficiency, mainly due to its large organizational overhead, but also because the efficient production of knowledge is often not its only (or even primary) goal. All of this implies that merely encouraging (or even enforcing) inclusive and open practices, while generating technological platforms and tools to implement them, will not be sufficient to propel citizen science beyond its current status as a specialized niche product—often criticized, belittled or ignored by commentators and academic researchers for its lack of rigour and efficiency. This is a serious problem, which is philosophical down to its core, and therefore calls for a philosophical solution. In order for citizen science to succeed beyond its current limitations, we need a fundamental reexamination of the nature and purpose of scientific knowledge, and how it is produced. In particular, we need to move beyond our increasing obsession with productivity metrics in science. Simply put, we require a new model for doing research, with corresponding procedures for quality control, that is more tolerant and conducive to diversity and inclusive participation (see also [12,29]).

**Figure 1.**
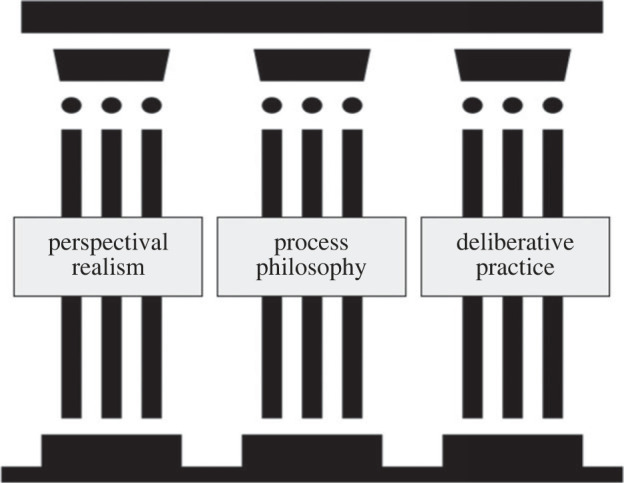
The three pillars of our ecological model for scientific research. See text for details.

In what follows, we outline an epistemology of science, which is formulated explicitly with our discussion of democratic citizen science in mind. It is centred around three main philosophical pillars (figure 1). The first is *perspectival realism* (also called *scientific perspectivism*), providing an alternative to naive realism which is appropriate for the twenty-first century [30,31]. The second is *process philosophy,* in the form of *naturalistic epistemology,* which focuses our attention away from knowledge as the product, or final outcome, of scientific research, towards the cognitive processes underlying knowledge production [32–34]. The third and final pillar is *deliberative practice*, with its focus on social interactions among researchers, which yields the surprising insight that we should not always reach for consensus in science [35]. These three pillars tightly intertwine and combine into a new model, which we could call *the ecological model of doing science,* because—just like an ecosystem—it is centred around diversity, inclusion, interaction, self-organization and robustness, in addition to (long-term) productivity. This model is based on a completely different notion of accountability, leading to process-oriented, participatory, and integrated assessment strategies for scientific projects that go far beyond any predefined narrow set of metrics to measure research output. We highlight specific procedures that enable us to adaptively monitor how these epistemic pillars are applied, and conclude our discussion with a number of concrete suggestions on how to implement such strategies in practice.

## 2. Naive realism and the cult of measurable productivity

What we mean here by naive realism is a form of *objectivist realism* that consists of a loose and varied assortment of philosophical preconceptions that, although mostly outdated, continue to shape our view of science and its role in society. The central tenet of naive realism is that the main (and only) epistemic goal of science is to find objective and universal Truth. The ideas supporting this popular notion are drawn from three main historical sources: the logical positivism of the Vienna Circle, Popper's falsificationism (somewhat ironically, as we shall see), and Merton's sociology of science.

Positivism in general, and *empirical* or *logical positivism* in particular, hold that information derived from sensory experience, interpreted through reason and logic, forms the source of all well-founded knowledge (e.g. [36–38]). The logical positivists asserted that meaningful discourse is either purely analytic (in the formal sciences, such as logic and mathematics) or empirically testable (in the natural and social sciences). Everything else is cognitively meaningless, in particular what became labelled as ‘metaphysics’: abstract philosophical theory that has no basis in reality. This is still reflected in the ‘I have facts, and therefore do not need any philosophy’ attitude of many current-day scientists.

At the heart of positivism lies *the principle of verification*: scientific hypotheses are positively confirmed by empirical evidence, which comes in the form of condensed summaries of direct observations, where all terms are defined ostensively, i.e. in an obvious and unambiguous manner. This firmly anchors scientific knowledge in objective reality, but it demands an exceptional degree of clarity, detachment, and objectivity on the part of the observer. The fact that human beings may not be able to achieve such detached, objective clarity was acknowledged by several logical empiricists themselves. Even our most basic observations are coloured by mood and emotions, biased assumptions, and all the things we already know.

In the meantime, Karl Popper—probably the world's best-known philosopher of science—revealed an even more serious and fundamental problem with verification: he showed that it is impossible, amounting to a logical fallacy (an affirmation of the consequent; e.g. [37,39]). By contrast, Popper argued that it *is* possible to *falsify* hypotheses by empirical evidence. Therefore, the only way to empirically test a scientific conjecture is to try to refute it. In fact, if it is not refutable, it is not scientific. This part of Popper's argument still stands strong today, and because of it (logical) positivism has become completely untenable among philosophers of science.

The doctrine of *falsificationism* may be the most widely held view of science among practising researchers and members of the wider public today. However, unknown to most non-philosophers, it has a number of problems and some very counterintuitive implications. First of all, falsificationism is completely incompatible with positivism, even though both views often coexist in the minds of naive realists. In Popper's view, scientific hypotheses stand as long as they have not yet been falsified, but they are never confirmed to be true in the sense of accurately reflecting some specific aspect of reality. Popper called this state on non-refutation *verisimilitude*, which literally translates as ‘the appearance of being true’. Furthermore, falsificationism provides a rather simplistic account of how science actually works. In practice, scientific theories are rarely discarded, especially not if viable alternatives are lacking. Instead of refuting them, theories are often amended or extended to accommodate an incompatible observation. Quite often, scientists do not even bother to adjust their theories at all: paradoxical results are simply ignored and classified as outliers. ‘Good enough’ theories often remain in use, even if better ones are available, as is the case in space exploration, which largely relies on Newtonian rather than relativistic mechanics. Finally, falsificationism has nothing to say about how hypotheses are generated in the first place. It turns a blind eye to the sources of scientific ideas, which remain a mystery, beyond philosophical investigation. In other words, it is deliberately ignoring the cognitive and social practices that are generating and processing scientific knowledge. Seen from this reductive angle, the creative aspects of science seem rather haphazard, even irrational, and the main role of the scientific method is a negative one: to act as a selection mechanism which objectively filters out yet another wrong or idiosyncratic idea.

On top of a fluctuating mix of positivist and Popperian ideas, naive realism often incorporates a simple *ethos of science* that goes back to the work of sociologist Robert K. Merton [40]. This ethos is based on four basic principles: (1) *universalism*—criteria to evaluate scientific claims must not depend on the person making the claim; (2) *communism* (or *communality,* for our American readers)*—*scientific knowledge must be commonly owned once it is published; (3) *disinterestedness—*scientists must disengage their interests from their judgements and actions; and (4) *organized scepticism—*scientific communities must disbelieve, criticize, and challenge new views until they are firmly established. According to Merton, scientists who conform to his ethos should be rewarded, while those that violate it should be punished. In this way, the ethos ensures that science can fulfil its primary societal role: to provide a source of certified, trustworthy, objective knowledge.

It should be evident—even from such a brief and cursory overview—that the ideas underlying naive realism do not form a coherent doctrine. Nor do they paint a very accurate picture of actual science, performed by actual human beings. In fact, the naive realist view is highly idealized: more about what science *should be like* in our imagination than about what it actually *is*. It provides a deceptively simple epistemological framework for an ideal science whose progress is steady, predictable, under our control. This is probably why it is still so attractive and influential, even today. Everybody can understand it, and it makes a lot of intuitive sense, even though it may not hold up to closer scrutiny. Its axiomatic nature provides an enticing vision of a simpler and better world than the complicated and imperfect one we actually live in. However, because of its (somewhat ironic) detachment from reality, there will likely be unintended consequences and a lack of adaptability if we allow such an overly simplistic vision to govern our way of measuring the success of science. Let us highlight some of the specific features of naive realism that lead to unforeseen negative consequences in science today.

**Figure 2.**
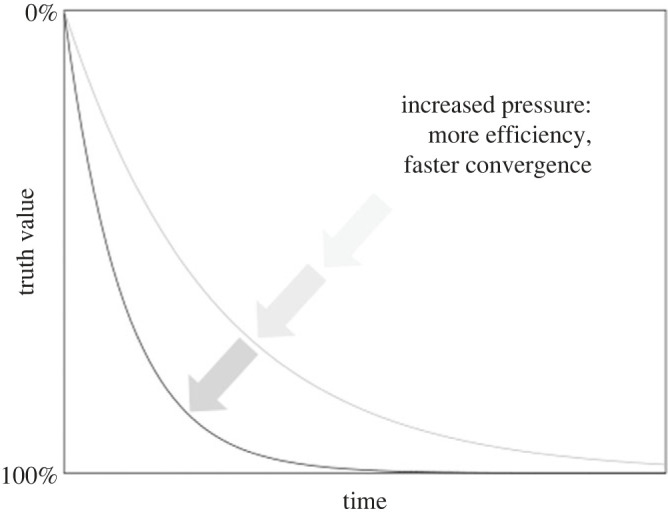
Naive realism suggests that the universal scientific method leads to empirical knowledge that approximates a complete understanding of reality asymptotically (represented by exponential functions in this graph). Scientific progress does not depend in any way on the backgrounds, biases, or beliefs of researchers, which are filtered out by the proper application of the scientific method. According to this view, simply applying increased pressure to the research system should lead to more efficient application of the scientific method, and hence to faster convergence to the truth. See text for details.

First of all, naive realism suggests that there is a single *universal scientific method*—based on logical reasoning and empirical investigation—which is shared by researchers across the natural and social sciences. This method allows us to verify, or at least falsify, scientific hypotheses in light of empirical evidence, independent of the aim or object of study. Considered this way, the application of the scientific method turns scientific inquiry itself into a mechanism, a purely formal activity. It works like an algorithm. If applied properly, scientific inquiry necessarily leads to *an ever-increasing accumulation of knowledge that approximates reality asymptotically* (figure 2). Because of our finite nature as human beings, we may never have definitive knowledge of reality, but we are undoubtedly getting closer and closer.

Complementary to this kind of formalization, we have a universally accepted ethos of science, which provides a set of standards and norms. When properly applied, these standards and norms guarantee the *validity and objectivity of scientific knowledge*. Scientific method and practice become self-correcting filters that automatically detect and weed out erroneous or irrational beliefs or biases. In that sense, scientific inquiry is seen as independent of the identity or personality of the researcher. *It does not matter who applies the scientific method*. The outcome will be the same as long as its standards and norms are followed correctly. All we have to do to accelerate scientific progress is to crank up the pressure and increase the number of scientists (figure 2).

This view has a number of profound implications:

— It sees researchers (once properly trained to adhere to the scientific method and ethos) as completely replaceable.
— It therefore fails to appreciate the diversity in researchers' experiences, motivations, interests, values, and philosophical outlooks.
— It leads to the idea that scientific inquiry can be optimized based solely on quantitative measurement of the productivity of individual researchers.

It is easy to see that all of these points are highly problematic, especially when considered in the context of democratic citizen science. Thus, a naive realist is better off without democratic citizen science, since there is no point in valuing the individual's motivations and point of view, there is no advantage in the diversity of citizen scientists, and it makes no sense to take into account a multiplicity of epistemic and non-epistemic goals beyond the efficient production of research output. All of these only lead to a loss of focus and slow traditional science down. Or do they?

In reality, the simplistic view of naive realism outlined above leads to *a* veritable *cult of measurable productivity* [26], which is steering science straight into a game-theoretical trap. The short-term thinking and opportunism that is fostered in a system like this, where rare funding opportunities confer a massive advantage and heavily depend on a steady flow of publications with high visibility, severely limits creative freedom and prevents scientists from taking on high-risk projects. Ironically, this actually *diminishes* the productivity of a scientific community over the long term, since the process of scientific inquiry tends to get stuck in local optima within its search space. Its narrow focus on short-term gains lacks the flexibility to escape.

What we need to prevent this dilemma is a less mechanistic approach to science, an approach that reflects the messy reality of limited human beings doing research in an astonishingly complex world [31]. It needs to acknowledge that there is no universal scientific method. Scientific research is a creative process that cannot be properly formalized. Last but not least, scientific inquiry represents an evolutionary process combining exploitation with exploration that thrives on diversity (of both researchers and their goals). Not just citizen science, but science in general, deserves an updated epistemology that reflects all of these basic facts. This epistemology needs to be taught to scientists and the public alike, if we are to move beyond naive realism and allow democratic citizen science to thrive.

## 3. Science in perspective

The first major criticism that naive realism must face is that there is no formally definable and universal scientific method. Science is quite obviously a cultural construct in the weak sense that it consists of practices which involve the finite cognitive and technological abilities of human beings, firmly embedded in a specific social and historical context. Stronger versions of *social constructivism*, however, go much further than that. They claim that science is *nothing but* social discourse (see [41] for an historical overview). This is a position of *relativism*: it sees scientific truths as mere social convention, and science as equivalent to any other way of knowing, like poetry or religion, which are simply considered different forms of social discourse. We find this strong constructivist position unhelpful. In fact, it is just as oversimplified as the naive realist stance. Clearly, science is neither purely objective nor purely culturally determined.

*Perspectival realism* [30,31,42] and, similarly, *critical realism* [43] provide a middle way between naive objectivist realism and strong forms of social constructivism. Both of these two flavours of non-naive realism hold that there is an accessible reality, *a causal structure of the universe*, whose existence is independent of the observer and their effort to understand it. Science provides a collection of methodologies and practices designed for us to gain trustworthy knowledge about the structure of reality, minimizing bias and the danger of self-deception. At the same time, perspectival realism also acknowledges that we cannot step out of our own heads: it is impossible to gain a purely objective ‘view from nowhere’ [44]. Our access to the world, at all levels—from the individual researcher to the scientific community to the whole of society and humanity—is fundamentally biased and constrained by our cognitive and technological abilities, which we exercise under particular social and historical circumstances.

Each individual and each society has its unique perspective on the world, and these perspectives *do* matter for science. To use Ludwik Fleck's original terms, every scientific community is a Denkkollektiv (*thought collective*) with its own Denkstil (*thought style*), which circumscribes the type and range of questions it can ask, the methods and approaches it can employ, and the kinds of explanations it accepts as scientific [45]. All of these aspects of inquiry have changed radically, time and again, throughout the history of philosophy and science, the most famous example being the transition from Aristotelian to Cartesian and then Newtonian styles of inquiry during the Scientific Revolution (see the open-source book [46] for an excellent overview). Our Denkstil is likely to evolve further in the future. In other words, there is no way to define science, or the scientific method, in a manner which is independent of social and historical context. Scientific inquiry is not formalizable in this way, and it never will be.

At this stage of our argument, it is important to note that by ‘perspective’ we do not mean just any arbitrary opinion or point of view. Perspectivism is *not* relativism (see also [24]). Instead, perspectives must be justified. This is the difference between what Richard Bernstein [47] has called *flabby* versus *engaged pluralism*. In the words of philosopher William Wimsatt, perspectives are ‘intriguingly quasi-subjective (or at least observer, technique or technology-relative) cuts on the phenomena characteristic of a system’ [31, p. 222]. They may be limited and context-dependent. But they are also grounded in reality. They are not a bug, but a central feature of the scientific approach. Our perspectives are what connects us to the world. It is only through them that we can gain any kind of access to reality at all [48]. Popper was right in saying that it is impossible to obtain absolutely certain empirical facts. Our knowledge is always fallible. But we can still gain empirical knowledge that is sound, robust, and trustworthy, at least up to a certain degree [31,49]. In fact, science gives us knowledge of the physical world that is *more* robust than what we get from other ways of knowing. That is precisely its purpose and societal function. Let us elaborate a bit more.

If scientific inquiry is not a purely formal activity, then scientific methods do not work like algorithms which are guaranteed to yield an ever-closer approximation to reality, no matter who is using them. Real science, performed by real scientists, does not actually aim to come up with a perfect explanation of everything, the whole of reality. Instead, researchers make use of *imperfect(ible) heuristics—*fallible short-cuts, improvisations that solve scientific problems (most of the time) in specific areas under specific circumstances [31,50]. Herbert Simon called this down-to-Earth approach to problem-solving *satisficing*, contrasting it to the largely unachievable optimal solutions sought by the naive realist [51,52]: heuristics may not be perfect, but they allow us to reach our epistemic goals within a reasonable amount of time, energy, and effort. This is an utterly pragmatic view of science.

Yet, science is not just problem-solving either. As Aristotle already recognized, the ultimate goal of inquiry is to supply us with a structured account of reality (see [32] for a contemporary discussion of this issue). This is possible, but not as easy as a naive realist might think. Being good at solving a particular problem does not automatically imply that a heuristic also teaches us something about the structure of reality. It could work for all the wrong reasons. How can we find out whether we are deceiving ourselves or not? In order to do this, we need to assess the *robustness* (or *soundness*) of the knowledge that a heuristic produces in a given context. Remember that empirical insights are never absolutely certain, but they can be robust if they are ‘accessible (detectable, measurable, derivable, definable, producible, or the like) in a variety of independent ways' [31, p. 196]. It *is* possible to estimate the relative robustness of an insight—what we could call *perspectival truth*—by tracking its invariance across perspectives, while never forgetting that the conditions that make it true always depend on our own present circumstances [49]. Thus, multiple perspectives enhance robust insight, and a multiplicity of perspectives is what democratic citizen science provides. It is by comparing such perspectives that science provides trustworthy knowledge about the world—not absolutely true, but as true as it will ever get.

Having multiple perspectives becomes even more important when we are trying to tackle the astonishing complexity of the world. Science is always a compromise between our need for simple-enough explanations to support (human) understanding, and the unfathomably complex causal structure of reality, especially in areas such as the life sciences (including ecology), or social sciences such as psychology, sociology and economics (e.g. [53]). Perspectival realism frees us of the naive realist idea that science must provide a single unified account of reality—a grand unified theory of everything. As a matter of fact, unified accounts are only possible for simple systems. By contrast, complex systems (in a perspectival sense) are *defined* by the number of distinct valid perspectives that can be applied to them [31]. A complex system is not just a complicated mechanism, like a clockwork or a computer. It can be viewed from many different angles, and none of these views provides all there is to know about the system. Nor do these views simply combine to form a complete or certain body of knowledge covering everything the system is capable of. The more such valid perspectives there are (and the less they simply add up), the more complex the system. Climate resilience is an excellent example of a scientific problem that is incredibly complex in this way, since a full understanding of its causes and consequences requires insights from a variety of actors (researchers, farmers, policy makers, technologists, and impacted populations), and from a variety of fields, ranging from biogeochemistry, ecology, agriculture and hydrology to economics and other social sciences. Without such diverse perspectives there can be no true understanding. Democratic citizen science can be an essential tool to provide more diversity, and thus more robustness in climate research.

Last but not least, diversity of perspectives lies at the very heart of scientific progress itself. Such progress can occur in two qualitatively different ways: as the ‘normal’ gradual accumulation and revision of knowledge, or in the form of *scientific revolutions* [54]. In this context, it is important to notice that when a new discovery is made, the resulting insight is never robust at first [31]. Its soundness must be gradually established. This is where Merton's universal scepticism reaches its limitations: if applied too stringently to new insights, it can stifle innovation. As a new insight becomes accepted, other scientific theories may be built on top of it through a process called *generative entrenchment* (ibid.). The more entrenched an insight, the more difficult it becomes to revise without bringing down the growing theoretical edifice that is being built on its foundation. For this reason, entrenched insights should ideally also be robust, but this is not always the case. Scientific revolutions occur when an entrenched but fragile insight is toppled [31,54]. Classic examples are the assumptions that space and time are pre-given and fixed, or that energy levels can vary continuously. The refutation of these two entrenched yet fragile assumptions led to the twin revolutions of relativity and quantum mechanics in early twentieth-century physics (see [46] for a recent review).

As we construct and expand our scientific knowledge of the world, more and more insights become robust and/or entrenched. At the same time, however, errors, gaps and discrepancies accumulate. The detection of patterns and biases in those flaws can greatly facilitate scientific progress by guiding us towards new problems worthy of investigation. Wimsatt [31] calls this the *metabolism of errors.* Basically, we learn by digesting our failures. For this to work properly, however, we need to be allowed to fail in the first place (see [55]). And, yet again, we depend on a multiplicity of perspectives. To detect biases in our errors, we require a disruptive strategy that allows us to ‘step out’ of our own peculiar perspective, to examine it from a different point of view. This is only possible if alternative perspectives are available. Scientific progress is catalysed by diversity in ways which a naive realist cannot even begin to understand.

In summary, we have shown that a diversity of perspectives is essential for the progress of science and for the robustness of the knowledge it generates. This diversity of perspectives, in turn, depends on the diversity of individual backgrounds represented in the communities involved in designing, managing and performing research. Of particular importance in this regard are individuals with a personal stake in the aims of a scientific project. Their perspectives are privileged in the sense of having been shaped by personal experience with the problem at hand, in ways which may be inaccessible to a neutral observer. Such engaged perspectives are called *standpoints* [56–58]*.* Each individual standpoint can broaden the scope and power of the cognitive and technological tools being brought to bear on an issue. This is particularly important in the context of climate resilience, where local experiences and challenges must be considered as an essential part of any problem solution. Being engaged (contra Merton's principle of disinterestedness) is desirable in this context, since it makes sure that proposed problem solutions are both applicable and relevant under a given set of particular conditions. In this way, democratic citizen science can become an essential tool for the production of adequate scientific knowledge. Therefore, it is of utmost importance that the relevant stakeholders are recognized and properly represented in the research process.

## 4. Science as process

The second major criticism that naive realism must face is that it is excessively focused on research outcomes, thereby neglecting the intricacies and the importance of *the process of inquiry*. Basically, looking at scientific knowledge only as the product of science is like looking at art in a museum. However, the product of science is only as good as the process that generates it. Moreover, many perfectly planned and executed research projects fail to meet their targets, but that is often a good thing: scientific progress relies as much on failure as it does on success (see §3). Some of the biggest scientific breakthroughs and conceptual revolutions have come from projects that have failed in interesting ways. Think about the unsuccessful attempt to formalize mathematics, which led to Gödel's Incompleteness Theorem [59], or the scientific failures to confirm the existence of phlogiston, caloric and the luminiferous ether, which opened the way for the development of modern chemistry, thermodynamics and electromagnetism, respectively [46]. Adhering too tightly to a predetermined research plan can prevent us from following up on the kind of surprising new opportunities that are at the core of scientific innovation. Research assessment that focuses exclusively on deliverables and outcomes, and does not integrate considerations about the process of inquiry, can be detrimental to scientific progress.

Sometimes, and especially in democratic citizen science, the goal *is* the journey. Democratic citizen science projects put a strong emphasis on facilitating their participants' individual learning, and their inclusion in the process of inquiry at the level of the research community (e.g. [60]). Furthermore, the problems of how to manage collaborations, data sharing and quality control are no longer peripheral nuisances, but themselves become a central part of the research focus of the project. Democratic citizen science is as much an inquiry into the natural world, as it is an inquiry into how to best cultivate and use humanity's collective intelligence (see [9]). The most valuable outcome of a citizen science project may very well be an improved learning and knowledge-production process. We now turn our attention to this dynamic. In this section, we look at the cognitive activities and research strategies that individual researchers use to attain their epistemic goals. The role of interactions among scientists and their communities will be the topic of §5.

The first thing we note is that scientific knowledge itself is not fixed. It is not a simple collection of immutable facts. The edifice of our scientific knowledge is constantly being extended [31]. At the same time, it is in constant need of maintenance and renovation (ibid.). This process never ends. For all practical purposes, the universe is cognitively inexhaustible (e.g. [33,61]). There is always more for us to learn. As finite beings, our knowledge of the universe will always remain incomplete. Besides, what we can know (and also what we want or need to know) changes significantly over time (e.g. [46]). Our epistemic goalposts are constantly shifting. The growth of knowledge may be unstoppable, but it is also at times erratic, improvised and messy—anything but the straight convergence path of naive realism depicted in figure 2.

Once we realize there is no universal scientific method, and once we recognize the constantly shifting nature of our epistemic goals, the process of knowledge production becomes an incredibly rich and intricate object of study in itself. The aim of our theory of knowledge must adapt accordingly. Classic epistemology, going back to Plato and his dialogue ‘Theaetetus’ [62], considered knowledge in an abstract manner as ‘justified true belief’, and tried to find universal principles which allow us to establish it beyond any reasonable doubt. This endeavour ultimately ended in failure (albeit an interesting one; e.g. [63,64]). *Naturalistic epistemology*, in contrast, goes for a more humble (but also much more achievable) aim: to understand the epistemic quality of actual human cognitive performance [32]. It asks which strategies we—as finite beings, in practice, given our particular circumstances—can and should use to improve our cognitive state: what are the processes that robustly yield reliable and relevant knowledge about the world? The overall goal of naturalistic epistemology is to collect a compendium of *cognitively optimal processes* that can be applied to the kinds of questions and problems humans are likely to encounter. This is a much more modest and realistic aim than any quixotic quest for absolute knowledge, but it is still extremely ambitious. Like the expansion of scientific knowledge, it is a never-ending process of iterative and recursive improvement—an ameliorative instead of a foundationalist project (ibid.). As limited beings, we are ultimately condemned to build on the imperfect basis of what we have already constructed.

Just like scientific perspectivism, naturalistic epistemology leads to context-specific strategies that allow us to attain a set of given epistemic goals. What is important in the context of our discussion is that different cognitive processes and research strategies will be optimal under different circumstances. There is no universally optimal search strategy for inquiry (or anything else)—there is no free lunch [65]. What approach to choose depends on the current state of knowledge and level of technological development, the available human, material and financial resources, and the epistemic goals of a project. These goals may be defined in terms of solving a particular problem, in terms of providing new insights into the structure of reality, and/or in terms of optimizing the research process itself. Choice of strategy is in itself an empirical question. Naturalistic epistemology must be based on history and empirical insights into error-prone heuristics that have worked for similar goals and under similar circumstances before [32]. We cannot justify scientific knowledge in a general way, but we can get better at appraising its epistemic value by studying the process of inquiry itself, in all its glorious complexity.

One central insight from this kind of epistemology, which is supported by empirical and theoretical evidence, is that evolutionary search processes such as scientific inquiry are subject to what Thomas Kuhn [66] has called *the essential tension* between a productive research tradition and risky innovation. This classical view in the philosophy of science has since been recast in computer science and popularized as the strategic balance between *exploration* (gathering new information) and *exploitation* (putting existing information to work) (for an accessible introduction, see chapter 2 of [67]). It is important to note, however, that we are not really talking about a balance in the sense of a static equilibrium here. The optimal ratio between the two strategies cannot be precisely computed for an open-ended process with uncertain returns such as scientific inquiry (ibid.). Instead, we need to switch strategy dynamically, based on local criteria and incomplete knowledge. The situation is far from hopeless though, since some of these criteria can be empirically determined. For instance, it pays for an individual researcher, or an entire research community, to explore at the onset of an inquiry. This happens at the beginning of an individual research career, or when a new research field opens up. Over time, as the field matures and information accumulates, exploration yields diminishing returns. At some point, it is time to switch over to exploitation. Imagine moving to a new city. Initially, you will explore new shops, restaurants and other venues, but eventually you will settle down and increasingly revisit your favourite places. This is an entirely rational meta-strategy, inexorably leading people (and research fields) to become more conservative over time (see [11,68–70] for evidence on this).

Here, we have an example where the optimal research strategy depends on the process of inquiry itself. A healthy research environment provides scientists with enough flexibility to switch strategy dynamically, depending on circumstances. Unfortunately, industrial science does not work this way. The fixation on short-term performance, measured through output-oriented metrics, has locked the process of inquiry firmly into exploitation mode (e.g. [71]). Put differently, exploration almost never pays off in such a system. It requires too much time, effort, and a willingness to fail. It may be bad for productivity in the short term, but is essential for innovation in the long run. This is the game-theoretic trap we discussed in §2. It is sustained by the narrow-minded view that the attainment of the epistemic goals of science can be accelerated simply by maximizing the rate of research output.

In this section, we have argued that naturalistic epistemology, an empirical investigation of the process of inquiry itself, could lead us out of this trap. But it is not enough. We also need a better understanding of the social dimension of doing science, which is what we will be discussing next.

## 5. Science as deliberation

The third major criticism that naive realism must face is that it is obsessed with consensus and uniformity. Many people believe that the authority of science stems from unanimity, and is undermined if scientists disagree with each other. Ongoing controversies about climate science or evolutionary biology are good examples of this sentiment (e.g. [2]). To a naive realist, the ultimate aim of science is to provide a single unified account—an elusive unified theory of everything—that most accurately represents *all* of reality. This kind of thinking about science thrives on competition: let the best argument (or theory) prevail. Truth is established by *debate*, which is won by persuading the majority of experts and stakeholders in a field that some perspective is better than all its competitors. As Robert Merton [40] put it: competing claims get settled sooner or later based on the principle of universalism. There can only be one factual explanation. Everything else is mere opinion.

However, there are good reasons to doubt this view. In fact, uniformity can be pernicious [35]. This is because all scientific theories are *underdetermined by empirical evidence*. In other words, there is always an indefinite number of scientific theories able to explain a given set of observed phenomena. For most scientific problems, it is impossible in practice to unambiguously settle on a single best solution based on evidence alone. Even worse: in most situations, we have no way of knowing how many possible theories there actually are. Many alternatives remain unconsidered [72]. Because of all this, the coexistence of competing theories need not be a bad thing. In fact, settling a justified scientific controversy too early may encourage agreement where there is none [35]. It certainly privileges the status quo, which is generally the majority opinion, and it suppresses (and therefore violates) the epistemic equality of those who hold a minority view that is not easy to dismiss (ibid.). In summary, too much pressure for unanimity leads to a dictatorship of the majority, and undermines the collective process of discovery within a scientific community.

Let us take a closer look at what this process is. Specifically, let us ask which form of information exchange between scientists is most conducive to cultivating and utilizing the collective intelligence of the community. In the face of uncertainty and underdetermination, it is *deliberation,* not debate which achieves this goal [35]. Deliberation is a form of discussion that is based on *dialogue*, rather than debate. The main aim of a deliberator is not to win an argument by persuasion, but to gain a comprehensive understanding of all valid perspectives present in the room, and to make the most informed choice possible based on the understanding of those perspectives (e.g. [73]). What matters most is not an optimal, unanimous outcome of the process, but the quality of the process of deliberation itself, which is greatly enhanced by the presence of non-dismissible minorities. As Popper already pointed out, the quality of a scientific theory increases with every challenge it receives. Such challenges can come in the form of empirical tests, or thoughtful and constructive criticism of a theory's contents. The deliberative process, with its minority positions that provide these challenges, is stifled by too much pressure for a uniform outcome. As long as matters are not settled by evidence and reason, it is better—as a community—to suspend judgement and to let alternative explanations coexist.

It is not difficult to see how deliberation—with its choice-making based on the understanding of multiple perspectives—is particularly important for interdisciplinary and transdisciplinary projects. Interdisciplinary projects are those in which scientists from different disciplines work together, while transdisciplinarity represents the most complex degree of cross-disciplinary collaboration, which aims to transcend the disciplinary boundaries within its domain altogether. Such projects boost scientific innovation when they manage to integrate different perspectives into a cohesive solution (reviewed in [68]). They help science break out of the inexorable tendency of research fields to become more conservative over time (see §4). They are key to generating and enhancing epistemic exploration. But, like other exploratory processes, they need time and effort to establish. Deliberative processes cannot be rushed. To integrate them into our research environment, we need to assess their quality directly.

Deliberative processes that facilitate collective intelligence work best with relatively small groups of deliberators, each with an engaged and non-dismissible standpoint on the matter at hand. However, many scientific projects—especially those of democratic citizen science—require human and material resources that go beyond the capabilities of small groups. This is particularly relevant in the field of climate resilience, where the number of impacted citizens reaches the planetary scale. In such cases, the deliberation process needs to be based on a suitable community structure in order to scale. This is why an increasing amount of science is done by teams [68]. There is empirical evidence that small teams of investigators are more innovative than isolated individuals or large-scale consortia [69]. This is because they strike a delicate balance between a diversity of standpoints and the ability of its members to productively engage in deliberation. The deliberative process can then be rescaled as an interaction between teams, resulting in a hierarchy of interactions that enable collective intelligence at multiple levels. This is an area of investigation that needs much more attention than it currently receives.

## 6. An ecological vision for citizen science

In §§3–5, we have outlined the three main pillars of an emerging epistemology that is tailored to the needs of democratic citizen science, but is equally applicable to academic research in general. We see the kind of citizen science it envisions as paradigmatic for a more participatory research environment, adequate for the complex planetary-scale problems humanity is facing today. Its highest aim is to foster and put to good use the collective intelligence of humanity. In order to achieve this, *we need research communities that are diverse, engaged, representative, and democratic*. What we propose here is an ‘ecological’ vision for a science which supports diversity, inclusion, and deliberation. This vision stands in stark contrast to our current industrial model of doing science (see §1). The two approaches are compared in table 1. Note that both models are highly idealized. They represent different ideals of how research ought to be done—two alternative ethos for science.

**Table 1.** Two idealized models for scientific research. This table compares different emphases exhibited by ‘industrial science’ versus ‘ecological science’. Note that both visions represent ideals, which are rarely attainable in practice. Most scientific projects will come to lie somewhere along the spectrum between these two extremes. See text for details.

‘industrial science’	‘ecological science’
control/prediction	participation/understanding
maximized short-term efficiency/output	maximized long-term productivity/robustness
competitive and closed	open and collaborative
intellectual monoculture	diversified perspectives
risk averse/exploitative	open to exploration

We have argued that the naive realist view of science is not, in fact, realistic at all. In its stead, we have presented an epistemology that adequately takes into account the needs and capabilities of limited human beings, solving problems in a world of planetary-scale interconnected complexity. The ecological research model proposed here is less focused on direct exploitation, and yet it has the potential to be more productive in the long term than the current industrial system. However, its practical implementation will not be easy, due to the game-theoretic trap we have manoeuvred ourselves into (see §2). Escaping this trap requires a deep understanding of the social and cognitive processes that enable and facilitate scientific progress for all. Finding such processes is an empirical problem, which is only beginning to be tackled and understood today. The argument we are making here is that such empirical investigations must be grounded in a suitable epistemological framework, and a correspondingly revised ethos of science, able to provide philosophical and ethical guidance for our attempts to improve our methods for scientific project management, monitoring, and evaluation through experience and experimentation. These methods must acknowledge the contextual and processual nature of knowledge production. They need to focus directly on the quality of this process, rather than being fixated exclusively on the outcome of scientific projects. They need to encompass multiple levels—from the individual investigator to their research community to the context of society in general. And they need to account for a diversity of epistemic goals.

Unfortunately, such explorative efforts are likely to fail unless we break out of the restrictive framework we have built around ourselves through an ever stronger focus on measuring research output, detached from any consideration of the cognitive and deliberative processes that generate it. Before we can achieve anything else, we must use our new appreciation of the process of inquiry to move beyond metric fixation, beyond the cult of productivity [26]. As a first step, this requires a broader awareness of the underlying philosophical issues. While the epistemological arguments we have presented here are well known among philosophers of science, they are virtually unheard of among practising scientists, science stakeholders and the general public. This urgently needs to change before we can have the kind of conversations that lead to sustainable changes in mindset and policy. Democratic citizen science is one of the most important initiatives towards increasing diversity, representation, and participation in science today. In addition, it is one of the main sources for new insights into the process of inquiry, and its process-oriented assessment. For these reasons, citizen science must play a key role in the upcoming transition from an industrial to an ecological model of doing research. In the final section of our paper, we will discuss the kind of measures we could experiment with to improve the assessment of citizen science projects along the lines of the philosophical argument we have presented above.

## 7. Beyond metric fixation: implications for project evaluation

**Figure 3.**
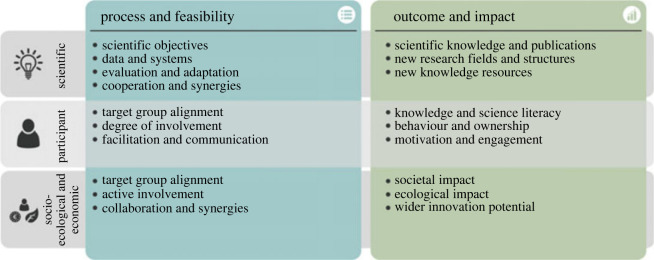
An assessment framework for democratic citizen science. Reprinted with permission from [28].

Our philosophical analysis points to a central conclusion: any proper evaluation of a scientific project must include *an epistemic appraisal of its process of inquiry*, including an assessment of the material, cognitive, deliberative, and organizational practices involved in knowledge production. It is not enough to judge a project by its outcome alone—the number of scientific publications it has produced, let us say, or the amount of factual knowledge its participants are able to regurgitate at a final debrief or exam. This central insight also underlies a recently proposed multidimensional evaluation framework for citizen science projects, which makes a fundamental distinction between process-based and outcome-based aspects of assessment [27,28]. It identifies three core dimensions to citizen science: scientific, participant, and socio-ecological/economic. For each of these, it defines criteria of evaluation concerning *both* aspects of ‘process and feasibility’ as well as ‘outcome and impact’ (figure 3). Such a framework can be applied not only to strategic planning, the selection of specific projects to be funded, and impact assessment after a project is finished, but also to monitor and, at the same time, to mentor participants and facilitate the progress of a project while it is running. Evaluation itself becomes a learning process—learning about learning—that supports participatory self-reflection and adaptive management practices [28].

Due to the epistemological nature of our argument, we focus mainly on the scientific knowledge dimension of this evaluation framework here, although epistemic processes underlying individual and collective learning and their wider societal and ecological impact are also subjects highly deserving of closer philosophical attention. In this context, it is worth repeating that not all citizen science projects have their main focus on the production of novel scientific theories, or the fundamental revision of existing scientific frameworks. Some are geared toward applied knowledge, or efforts in community-level data collection. Moreover, non-epistemic goals—changes in individual attitudes and behaviour, cultural practices or policies, for example—can be equally or even more important in some cases. For this reason, the evaluation framework in figure 3 is designed to be flexible and adaptive in terms of weighting different criteria. Moreover, while we limit our discussion to process-based aspects of scientific knowledge production, we do not want to leave the impression that evaluation of outcome is unimportant. *Both* aspects need to be considered together. What we do want to do here is to highlight the fact that process-based assessment remains undervalued, underdeveloped, and underused in the current system of academic research. Our analysis provides epistemological reasons for addressing this problem. Developing adequate approaches to process-based assessment requires an improved understanding of suitable practices of individual and community-level knowledge production that can actually be carried out in today's research environment.

Beyond emphasizing processual and participatory methods of evaluation, there is another fundamental point that arises from our analysis: many of the features that make democratic citizen science (and science in general) worthwhile and productive are impossible to capture by performative metrics. For example, the originality, relevance, and value of a scientific insight cannot be quantified objectively, because notions of ‘originality’, ‘relevance’ and ‘value’ contain fundamentally subjective and radically context-sensitive facets that are crucial to their meaning. Similarly, there is no standardized algorithm to assess the robustness or soundness of a piece of scientific knowledge. Instead, proper robustness analysis requires a careful comparison of scientific perspectives and an assessment of their independence from each other, which cannot be done without deep insight into the research topic and all the approaches that are being compared [31]. Standardized measures can support, but never fully replace judgement based on experience. Similarly, there is no metric for the generalizability or the adaptiveness of a scientific result. The range of circumstances under which some theory or insight may be usefully applied is impossible to predict, or even prestate [61,74]. Discovery cannot be planned in this sense. Much of scientific inquiry is driven by serendipitous coincidences, historical accidents, which cannot be captured by any predictive measure based on past evidence alone.

Thus, discovery cannot be forced, but it can be facilitated by providing an environment that is conducive to it. Our epistemological framework implies that this can be achieved by incentivizing collaborative processes and deliberation based on a diversity of standpoints. Obviously, this same argument also applies to the assessment of the wider socio-ecological implications of a project, its stakeholder engagement, its social embeddedness, and so on [28]. Each research project should be assessed under consideration of its particular scientific and societal circumstances, as well as its particular epistemic and non-epistemic goals. Even so, much of its value will only become evident in hindsight. Trying to define one-size-fits-all metrics or numerical indicators for qualities such as originality, relevance, robustness, adaptedness, or generalizability is bound to be counterproductive, because each and every scientific project, and the knowledge it generates, is different. Generalized abstraction ignores situation-dependent nuances, which may be essential for the success of a project and can only be assessed qualitatively and in retrospect.

Finally, there is another problem that arises in systems where rewards and punishments no longer depend on professional judgement—based on personal experience, honesty, dedication, and talent—but on quantitative indicators implemented as standard metrics of comparative performance. Such systems become vulnerable to *metric gaming* [26]. When a metric becomes the target of the measured system, Goodhart's Law applies, which states that such metrics are no longer good indicators for the system's original purpose. Efforts become channelled into optimizing performance as measured by the metric, often in ways that are not conducive to the system's original goals. This happened, for example, to the US school system after the introduction of standardized testing, which led to widespread teaching to the test (ibid.). Similarly, surgeons who are rated on the number of their successful operations often refuse to take on difficult cases (ibid.).

Metric gaming is also taking over the academic research system, where an unhealthy fixation on publication metrics leads to risk avoidance and the short-term optimization of personal research output to the detriment of community-level, long-term progress. Somewhat ironically, this trend *is* measurable: while the content of individual scientific publications is progressively diminishing, approaching what has been called the minimal publishable unit of information, the number of authors per paper is rapidly increasing (e.g. [75]). These trends are empirical signs of an academic system that is being systematically manipulated. Such a system no longer rewards those who do the best work, but those who are most efficiently gaming the metric, and hence the reward system in general.

All of this poses a formidable challenge for scientific project evaluation. On the one hand, we really do need methods to compare the quality of scientific projects: how else are we going to implement a fair and rigorous system for strategic planning, funding, monitoring, and assessment in research? On the other hand, we know that the value of a scientific project is radically context-dependent, and that standardized metrics make a system vulnerable to being gamed. As we have seen in §3, this does not necessarily have to lead us into relativism, considering any project as good as any other. There *are* criteria by which we can assess the promise and importance of a project, or the robustness of the knowledge it produces. What we need then, if we want to adopt an ecological model of citizen science, is an approach to evaluation, grounded in a perspectival, naturalistic, deliberative epistemology that is flexible and adaptable to the specific needs and circumstances at hand, and yet rigorous in its approach to epistemic appraisal. An example of such an evaluation model was recently used to establish a community-driven review system for rapidly adaptive micro-grants [76].

We have said this before, but it bears repeating: the essential first step towards such an approach is to overcome our current metric fixation [26]. Instead of being based on a set of fixed standards and metrics, project assessment ought to be grounded on shared values and procedures, themselves constantly subject to evaluation. To attain that goal, scientific assessment should not only evaluate the quality of the deliberative process of inquiry, but must itself become a deliberative, participatory, and democratic process.

Second, we need to carefully choose appropriate procedures to evaluate both quantifiable and non-quantifiable aspects of a project, and how they compare with alternative approaches in terms of achieving its specific goals. These procedures should be adapted to context, transparent, flexible, and they should include an element of self-evaluation. One suitable model is *co-evaluation* [77], an approach to assessment that includes all actors involved in or affected by a project in an iterative process and is based on methods from participatory action research (e.g. [78–80]). On top of this, there must be a meta-level process that evaluates the evaluators as they assess a project, guided by deliberative procedures. Finally, assessment must include an evaluation of the quality of this deliberative process itself. More than resembling the hierarchical mechanism of a clockwork, this method of project assessment imitates the self-regulatory and homeostatic dynamics of a living organism.

## 8. Conclusion

In this paper, we have introduced an epistemological framework that can serve as the foundation for the development and adaptation of new descriptors and procedures for project evaluation in democratic citizen science and academic research in general. This framework is based on the three pillars of perspectival realism (§3), process thinking in the form of naturalistic epistemology (§4), and deliberative practice (§5), leading to what we have called an ‘ecological model’ of doing research (§6). Perspectivism implies that the range of backgrounds and motivations of individual researchers in a community greatly influences the kind of questions that can be asked, the kind of approaches that can be used, and the kind of explanations that are accepted in a given research and innovation field. Naturalistic epistemology focuses our attention on the quality of the cognitive processes leading to a given research output, while deliberative practice emphasizes the community-level social dynamics that are required to enable collective intelligence. Together, these pillars lead to a new research ethos that values diversity, inclusion, and good communication much more than the traditional Mertonian approach to science (see §§2 and 6).

We have described the implicit amalgamation of positivist, Popperian and Mertonian ideas in the minds of scientists and stakeholders as ‘naive realism’ (§2). It could be argued, though, that our own vision of democratic citizen science is itself naive. In fact, Mirowski [4] has characterized open science (and citizen science with it) as something even worse: a pretext to extend neoliberal free-market thinking, with the aim of enabling platform capitalism (as exemplified by online giants such as Google and Facebook, or publishing corporations such as Elsevier) to build commercial monopolies from the systems of knowledge production. We are sympathetic to Mirowski's criticism, but emphasize that what he describes is a citizen science as it exists (and struggles) in the current status quo of the industrial system. Our attempt to sketch a more ‘ecological’ epistemological framework for academic research could be seen as an attempt to provide the philosophical foundations for the new ‘political ontology’ and the ‘economic structure’ Mirowski is calling for (ibid.). We are in no way naive enough to think this will be easy to implement under the current socio-political circumstances, or that it will be achieved in some sort of utopian way. Instead, we see the new ethos of science we are outlining here as something that can guide and inspire us while working pragmatically towards a more humane and sustainable research system based on more democratic values and procedures.

The main feature of our ecological model of research—what makes it resilient towards attempts at gaming the rules—is its adaptive flexibility: it adjusts itself to the circumstances of each project to be evaluated—its epistemic and non-epistemic aims, the backgrounds and motivations of its participants, and the nature of its particular research question and methodology. It employs a situated process-based quality assessment that relies on shared values and procedures, rather than standard metrics (which may still be used to support it, of course, but are no longer the only evaluative tool). Its adaptive nature renders it more resilient against attempts at gaming the system. The assessment process becomes a learning process itself, which can dynamically react to novel circumstances and challenges (see §7).

Our framework requires that we pay much more attention to the process of inquiry than in a traditional system, where evaluation is largely based on immediate and measurable research outcome. In particular, *we recommend quality assessment to focus on the aspects of diversity, inclusion, and deliberation*. The evaluation of the potential of a project should be combined with constant monitoring and facilitation of the research process. Are all relevant standpoints of impacted stakeholders represented in the community? Do project participants feel they are heard and can make a relevant contribution to the project? Is the deliberative format properly facilitated? Does it enable high-quality cognitive engagement of participants with the research problem at hand? Do participants understand the ethos of doing scientific research and innovation? Do they understand the criteria by which they will be evaluated? Are they given enough autonomy? Are they allowed to fail, while still having their efforts appreciated? Can they disagree with the majority view during deliberation? Can they comment on and contribute to the evaluation of their efforts themselves? This kind of process-focused assessment and facilitation allows a project to be deemed a success, if its process was properly implemented, even if the desired output may not have materialized at the end of the project. It allows participants and evaluators to jointly learn from their successes and (often more importantly) from their failures. And it generates a more collaborative and positive atmosphere in which to undertake creative work. Such a system cannot compete with industrial science on short-term efficiency. It takes time and effort to implement, and the deliberative process is optimized for participation and learning, rather than production. In the long run, however, this system has the potential to be more productive and innovative than the present one. It provides a way for exploration to reenter the world of academic research, allowing us to escape the local search maxima that the game-theoretic trap of the cult of productivity has gotten ourselves stuck on.

## Data Availability

This article has no additional data.
